# Physical activity and sedentary behavior levels among individuals with mental illness: A cross-sectional study from 23 countries

**DOI:** 10.1371/journal.pone.0301583

**Published:** 2024-04-26

**Authors:** Fernanda Castro Monteiro, Felipe de Oliveira Silva, Aline Josiane Waclawovsky, José Vinícius Alves Ferreira, Fabianna Resende de Jesus-Moraleida, Felipe Barreto Schuch, Philip B. Ward, Simon Rosenbaum, Rachel Morell, Lara Carneiro, Andrea Camaz Deslandes

**Affiliations:** 1 Psychiatry Institute, Federal University of Rio de Janeiro, Rio de Janeiro, Brazil; 2 Federal University of Santa Maria, Santa Maria, Brazil; 3 Graduate Program in Physiotherapy and Functioning, Department of Physiotherapy, Federal University of Ceará, Fortaleza, Brazil; 4 School of Psychiatry, UNSW Sydney, Australia and Schizophrenia Research Unit, Ingham Institute of Applied Medical Research, Liverpool, NSW, Australia; 5 Mindgardens Neuroscience Network, Sydney Australia Discipline of Mental Health and Psychiatry, UNSW Sydney Australia, Sydney, Australia; 6 Physical Education Department, College of Education, United Arab Emirates University, Al Ain, Abu Dhabi, United Arab Emirates; Hamasaki Clinic, JAPAN

## Abstract

People with mental illness tend to present low levels of physical activity and high levels of sedentary behavior. The study aims to compare these levels in mental illness patients, exploring the role of socioeconomic development and treatment setting. This cross-sectional study used accelerometers and the Simple Physical Activity Questionnaire (SIMPAQ) to assess physical activity and sedentary behavior in mental illness individuals living in 23 countries. Two-way ANOVAs were used to evaluate the interaction between socioeconomic development and the treatment settings on physical activity and sedentary behavior. A total of 884 (men = 55.3%) participants, mean age of 39.3 (SD = 12.8), were evaluated. A significant interaction between socioeconomic development and treatment settings was found in sedentary behavior (F = 5.525; p = 0.019; η2p = 0.009; small effect size). Main effects were observed on socioeconomic development (F = 43.004; p < 0.001; η2p = 0.066; medium effect size) and treatment setting (F = 23.001; p < 0.001; η2p = 0.036; small effect size) for sedentary behavior and physical activity: socioeconomic development (F = 20.888; p < 0.001; η2p = 0.033; small effect size) and treatment setting (F = 30.358; p < 0.001; η2p = 0.047; small effect size), showing that HIC patients were more active, while MIC patients were more sedentary. Moreover, despite of inpatients had presented higher levels of physical activity than outpatients, they also spent more time sitting. Socioeconomic development plays an important role in sedentary behavior in patients with mental disorders, warning the need to develop new strategies to reduce these levels in this population.

## Introduction

In 2019, approximately 12% of the global population had mental disorders [1]. Therefore, these individuals have a high burden of disability-adjusted life years and a high risk of premature mortality compared with the general population [2–5]. Premature mortality in this population is likely to be associated with high rates of physical morbidities, such as obesity, diabetes, and cardiovascular disease [6–8].

Previous evidence has shown high levels of sedentary behavior (SB) and low levels of physical activity (PA) among this population [9]. Despite efforts by public agencies, such as the World Health Organization (WHO), to reduce physical inactivity levels [10], the influence of socioeconomic factors on PA and levels of SB has received little attention and could play a role in this alarming scenario [11]. Most published population-based estimates of SB are limited to high-income countries (HIC) [12], which limits our ability to transfer these findings to those who live in areas where resources are scarce and/or have different social backgrounds.

Growing economic inequalities in lower-middle-income countries (LMIC) directly impact mental health [13]. According to the Global Burden of Disease (GBD) 2019, mental disorders are among the top ten leading causes of burden, with mental health services under-resourced in many countries and regions worldwide, especially in LMIC settings [14]. Despite efforts to minimize these disparities, only one-third of people with mental disorders are treated in some HIC, while, at worst, less than 5% of people living with mental illness from LMIC receive any treatment or care [13]. Disparities between LMIC and HIC have also been observed in PA and levels of SB [15]. A previous meta-analysis showed that people with severe mental illness in Europe tended to have higher levels of PA than those in South America [9]. However, the studies included in this meta-analysis used different tools to evaluate PA and SB. Therefore, there is a lack of studies that evaluate PA and SB in different countries using the same tools. In a study by Pogrmilovic et al. (2020) that involved 76 countries (the majority were HICs), 62% had national PA guidelines, while 40% had guidelines for SB [16]. The authors found significant differences in the availability of PA guidelines between country groups by income level and between regions of the world. Similarly, they found a significant difference in the availability of guidelines for SB between country groups by income level [16].

Despite efforts to implement PA as an adjuvant treatment in mental health systems, little is known about this approach in some MICs. The lack of priority given to exercise programs in these countries has resulted in an evidence gap, especially compared to studies involving HICs. Moreover, outpatients living in MIC face specific barriers. Environmental factors such as the lack of sidewalks, crosswalks, and safe places to exercise could result in additional barriers to achieving and maintaining physically active behavior [17]. Physical (physical illness, low energy, poor health), psychological (low motivation, self-confidence, and stress), and socio-ecological barriers (lack of time, support, and information) to exercise might also be observed in this population [18]. Therefore, due to the limited attention paid to the particularities of the treatment setting in MIC, novel strategies and programs must be considered to increase PA and decrease levels of SB. Vancampfort and colleagues showed that inpatients with severe mental illness were more physically active than outpatients (90.1 min per day vs. 32.5 min per day) [9]. In other studies, the authors found that PA levels were associated with a lower metabolic risk and beneficial changes in psychotic symptoms in both inpatients and outpatients with severe mental illness [19, 20]. In a recent meta-analysis involving inpatients with mental illness, Martland and colleagues found that physical exercise promoted a moderate decrease in depressive symptoms in all diagnoses (SMD: -0.416; 95%CI, -0.787 to -0.045; I^2^ = 79.69%) [21].

Another important limitation of knowledge in this area is the assessment of PA and SB. Individuals with mental illness tend to underestimate SB levels and overestimate PA levels in self-reported questionnaires. Although this type of assessment provides a simple way to collect data, it can present some limitations and bias, resulting in inaccuracies, especially in populations with mental illness. However, a systematic review and meta-analysis showed that subjective measures provided by self-reported questionnaires could be useful and reliable to assess PA levels in individuals with mental illness [22]. Meanwhile, objective measures, such as accelerometers, can provide accurate measures of PA and SB and are recognized as gold standard measurements [23].

However, there is a lack of studies investigating possible differences between treatment settings and their influence on subjective and objective measurements of PA and levels of SB, especially in the context of MIC.

In light of this, this study aimed to compare the relationship between socioeconomic development and treatment settings on PA and levels of SB in people with mental illness from HIC and MIC in different treatment settings. We hypothesized that income inequality and socioeconomic development are related to PA and SB. Specifically, we hypothesized that people receiving inpatient care would be more sedentary and less physically active than those receiving outpatient care. Regarding socioeconomic status, we expected individuals from MIC to be more sedentary and less physically active than those from HIC.

## Methods

This was a cross-sectional study of the relationship between socioeconomic development and treatment setting on PA and SB levels among people with mental illness from the Simple Physical Activity Questionnaire (SIMPAQ) study [24]. Specifically, the present study it was a secondary analysis of the original SIMPAQ database conducted between May 2021 and October 2022. The initial data collection from the original database was carried out between May 2016 and July 2017. The questionnaire was developed by a multidisciplinary team between 2014 and 2016. The validation was carried out in 2016 and 2017 in 23 countries. The countries included in this study were members of a multidisciplinary team. Eligibility criteria for potential sites included the willingness to recruit patients who met the inclusion criteria outlined below and the availability of a site coordinator with experience in research on mental health or physical activity. The SIMPAQ study protocol was approved by the Human Research Ethics Committee of the UNSW, Sydney, Australia (HC15586). Local ethics approval was obtained from each participating country according to local requirements. Each country in which a site acquired SIMPAQ data was assigned an income status based on the World Bank classification (www.worldbank.org).

### Study procedures

The participants were approached by a researcher nominated by the site coordinator who was not involved in the direct care of the patient. Written informed consent was obtained from all participants included in the study and they were willing to wear an accelerometer for 7 days. The eligibility criteria were as follows: 1) age between 18 and 65 years; 2) current inpatient or outpatient of one of the treatment facilities identified as a SIMPAQ validation study site; and 3) met the DSM-5 or ICD-10 criteria for any mental illness, excluding eating disorders. Data were collected in two face-to-face interviews conducted at least 7 days apart. Sociodemographic data, including symptoms and cognitive ability assessments, were collected. After the participants completed the SIMPAQ questionnaire (Time 1), they were provided with a triaxial accelerometer (ActiGraph GT3x or GT3x +). Seven days later, the accelerometer was removed, and the participant answered the SIMPAQ (Time 2). Individuals without a mental illness diagnosis and those without complete SIMPAQ or accelerometer data were excluded. The original SIMPAQ database included 1010 participants. In this study, 126 participants were excluded due to missing data from the SIMPAQ questionnaire and/or accelerometer measures.

#### Psychiatric diagnoses

Psychiatric diagnoses were obtained from medical records and initially evaluated by physicians. Participants may have met the criteria for more than one psychiatric diagnosis, and all diagnoses applied to each participant were recorded. The standardized form asked researchers to answer yes or no for the following diagnostic categories based on the clinical diagnosis: schizophrenia spectrum disorders, bipolar disorder, depressive disorder, anxiety disorder, obsessive-compulsive disorder, substance-related and addictive disorders, neurocognitive disorders, and other disorders. Individuals assigned to a single diagnostic category and those with psychiatric comorbidities were identified.

#### Symptom severity–DSM-5 self-rated level 1 cross-cutting symptom measure

The 23-item DSM-5 self-rated level 1 cross-cutting symptom measure was developed to assess the severity of symptoms in 13 psychiatric domains. These domains include depression, anger, mania, anxiety, somatic symptoms, suicidal ideation, psychosis, sleep problems, memory, repetitive thoughts and behaviors, dissociation, personality functioning, and substance use [25]. Each question was about the past 3 weeks, specifically, how much or how often the individual was affected by symptoms. The measurement was rated on a 5-point scale (0, none or not at all; 1, slight or rare, less than a day or two; 2, mild or several days; 3, moderate or more than half a day; and 4, severe or almost every day). The SIMPAQ study summed the total scores across these domains and dichotomized the scores around the median (20); lower symptom severity was defined as a score of < 21, and higher symptom severity was defined as a score ≥ of 21 [24].

#### SIMPAQ

The SIMPAQ is a self-report questionnaire aimed at evaluating the PA levels of an individual during the past 7 days. It has been translated into several languages, enabling its application and validation in different centers [24, 26]. For SIMPAQ active time, we assessed moderate to vigorous physical activity using the SIMPAQ questionnaire (box 3 + box 4). In this study, we call this variable SIMPAQ moderate to vigorous physical activity (MVPA).

#### Accelerometer (ActiGraph)

ActiGraph captures and records high-resolution raw acceleration data that are converted into a variety of objective activities and sleep measures using algorithms. Measures included gross acceleration, activity counts, energy expenditure, MET rate, steps taken, PA intensity, sedentary time, body position, sleep latency, total sleep time, awakening after sleep onset, sleep efficiency, ambient light, and heart rate intervals. The information of each participant was entered into CentrePoint, an online portal designed and distributed by Actigraph specifically to coordinate multi-site studies [27]. For the active time of the accelerometer, we used accelerometer MVPA measurements recorded by ActiGraph GT3x or GT3x +.

#### Statistical analysis

The Shapiro−Wilk test was used to verify the normal distribution of the data. Descriptive data were presented as means, and categorical variables were presented as absolute frequencies (%). Chi-square analysis was used to verify categorical variables. An independent t-test was performed to compare differences between the HIC and MIC groups in years of education, age, and body mass index (BMI). Kruskal−Wallis tests were performed to compare the groups (HIC × MIC, inpatient × outpatient) regarding SIMPAQ variables (PA and SB). Two-way ANOVA was used to verify the interaction between income status and living situation regarding accelerometer variables (PA and SB). Moreover, we included the partial eta-squared (*η*^*2*^*p*) and Cohen’s *f* effect size (*f*). Considering our study characteristics and the utilization of two-way ANOVA, the effect size of the partial eta-squared (*n*^*2*^*p*) is preferred to eta-squared. The magnitude of the effect size was interpreted as suggested by Cohen (1988) (*n*^*2*^*p*: 0.01, small; 0.06, medium; and 0.14, large; Cohen’s *f*: 0.10, small; 0.25, medium; 0.40, large) [28]. We used the Statistical Package for the Social Sciences (SPSS® 26.0 (Armonk, NY, USA) to perform the statistical analyses. The level of statistical significance was set at p ≤ 0.05.

## Results

### Demographic characteristics

A total of 884 individuals from 23 countries were included in this study. The total sample was predominantly composed of males (55.3%), from HIC (76.7%), and was recruited from an inpatient facility (53.5%). Considering the setting of the treatment and the sex of the total sample, males in inpatient care were 58%, while females were 42%. In outpatient care, males were 52% while females were 48%. Among individuals with a single diagnosis, the most prevalent condition in the total sample was schizophrenia (22.5%), followed by depression (17.5%) and bipolar disorder (16.1%). The mean number of psychiatric comorbidities was 32.7%. All descriptive data for the sample are presented in detail in Table 1.

**Table 1:** Demographic characteristcs.

	Total sample	HIC	MIC	*X^2^/t*	*p*
	(N = 884)	(N = 678)	(N = 206)		
**Treatment settings—N (%)**				11.484^b^	0.001*
Inpatient	473 (53.8)	384 (56.9)	89 (43.4)		
Outpatient	407 (46.2)	291 (43.1)	116 (56.6)		
**Sex—N (%)**				0.098^b^	0.755
Male	489 (55.3)	377 (55.6)	112 (54.4)		
Female	395 (44.7)	301 (44.4)	94 (45.6)		
**Sex/treatment setting–N (%)**				3.241^b^	0.072
Male inpatient	275 (58)	214 (55.7)	61 (68.5)		
Female inpatient	198 (42)	170 (44.3)	28 (31.5)		
Male outpatient	212 (52)	161 (55.3)	51 (44)		
Female outpatient	195 (48)	130 (44.7)	65 (56)		
**Education–N (%)**				6.829^b^	0.145
Never studied	3 (0.4)	3 (0.5)	0 (0.0)		
1–4 years	30 (3.7)	25 (3.8)	5 (3.1)		
5–8 years	96 (11.6)	68 (10.4)	28 (17.1)		
9–11 years	166 (20.2)	132 (20.1)	34 (20.7)		
> 12 years	526 (64.1)	429 (65.2)	97 (59.1)		
**BMI–mean (SD)**	27.06 (5.8)	27.17 (5.7)	26.68 (6.0)	1.11^a^	0.268
**Age–mean years (SD)**	39.3 (12.8)	39.5 (12.8)	38.5 (12.9)	0.70^a^	0.480
**Diagnosis—N (%)**				101.869^b^	0.001*
Psychiatric comorbidity	289 (32.7)	258 (38.1)	31 (15.0)		
Schizophrenia	199 (22.5)	172 (25.4)	27 (13.1)		
Depression	155 (17.5)	101 (14.9)	54 (26.2)		
Bipolar disorder	142 (16.1)	75 (11.1)	67 (32.5)		
Anxiety	35 (4.0)	29 (4.3)	6 (3.0)		
Substance disorders	34 (3.8)	22 (3.2)	12 (5.8)		
Obsessive compulsive disorder	15 (1.7)	8 (1.2)	7 (3.4)		
Trauma and stress	15 (1.7)	13 (1.8)	2 (1.0)		

a = independent t-test comparison between HIC and MIC—mean (standard deviation)

b = chi-square analysis—number (frequencies)

*p ≤ 0.05. HIC = high-income countries; MIC = middle-income countries; SD = standard deviation.

### Physical activity and sedentary behavior

Among countries, the most physically active was Switzerland, with a mean of 2.76 (SD = 1.2) hours/day of MVPA in accelerometer measures. The least physically active country was Denmark, with a mean of 0.35 (SD = 0.2) hours/day of MVPA (Table 2). In subjective measures, Japan was the most physically active, with a mean of 2.40 (SD = 1.5) hours/day of active time. The least physically active was Portugal, with a mean of 0.80 (SD = 0.5) hours/day (Table 2). In the total sample, participants had a mean of 1.15 (SD = 1.08) hours/day of MVPA in accelerometer measures and self-reported a mean of 1.33 (SD = 1.26) hours/day of active time in subjective measures (Table 3).

**Table 2:** Accelerometer and SIMPAQ measures in hours per day by country.

	Accelerometer MVPA	SIMPAQ MVPA	Accelerometer sedentary time	SIMPAQ sedentary time
	Mean (SD)	Mean (SD)	Mean (SD)	Mean (SD)
**HIC (N)**				
Australia (121)	0.68 (0.4)	1.26 (1.1)	10.77 (2.5)	8.47 (3.6)
Belgium (32)	0.89 (0.3)	1.13 (1.1)	10.91 (1.2)	7.43 (2.9)
Canada (28)	0.86 (0.6)	0.92 (0.8)	12.35 (2.6)	8.29 (3.5)
Chile (16)	1.03 (0.3)	1.00 (1.0)	9.48 (1.4)	8.81 (4.4)
Czech Republic (8)	0.97 (0.5)	1.24 (1.1)	12.92 (2.4)	7.42 (2.0)
Denmark (23)	**0.35**^**-**^ **(0.2)**	1.32 (1.1)	9.31 (1.0)	7.05 (3.7)
Germany (56)	0.93 (0.4)	0.85 (1.0)	11.10 (1.8)	7.76 (2.9)
Hong Kong (13)	0.90 (0.2)	0.99 (1.1)	11.41 (3.2)	8.87 (2.7)
Ireland (20)	1.18 (0.3)	1.18 (1.0)	10.94 (2.6)	7.57 (2.8)
Italy (100)	0.57 (0.4)	1.48 (1.3)	**8.33**^**-**^ **(1.3)**	7.44 (3.2)
Japan (18)	0.97 (0.4)	**2.40**^**+**^ **(1.5)**	11.42 (1.5)	8.35 (3.3)
Netherlands (13)	0.89 (0.8)	1.57 (1.4)	10.39 (0.8)	7.92 (4.3)
Norway (18)	0.75 (0.4)	1.48 (1.3)	11.17 (2.1)	**6.21**^**-**^ **(2.6)**
Portugal (11)	0.74 (0.3)	**0.80**^**-**^ **(0.5)**	8.96 (1.1)	**10.00**^**+**^ **(3.2)**
Spain (33)	0.98 (0.6)	1.52 (1.4)	10.79 (1.6)	8.89 (3.0)
Switzerland (131)	**2.76**^**+**^ **(1.2)**	1.52 (1.4)	13.12 (2.3)	7.83 (3.1)
Taiwan (27)	2.17 (1.0)	1.96 (1.4)	12.24 (1.8)	9.03 (3.6)
United States (10)	0.82 (0.2)	1.21 (0.9)	10.16 (1.2)	6.83 (4.0)
Total (478)	1.26 (1.1)	1.36 (1.2)	11.02 (2.5)	8.00 (3.3)
**MIC (N)**				
Brazil (37)	0.47 (0.3)	1.02 (0.9)	**14.24**^**+**^ **(2.4)**	8.00 (3.3)
India (42)	0.38 (0.3)	1.19 (1.3)	13.13 (2.6)	8.32 (3.5)
Iran (34)	1.84 (0.9)	1.05 (1.1)	13.80 (2.4)	8.50 (2.8)
Nigeria (10)	0.57 (0.1)	1.51 (1.8)	13.69 (2.5)	9.05 (2.8)
Pakistan (83)	0.46 (0.2)	1.36 (1.1)	10.10 (2.0)	8.62 (3.2)
Total (206)	0.73 (0.7)	1.22 (1.1)	12.72 (2.9)	8.45 (3.2)

SD = standard deviation; MVPA = moderate to vigorous physical activity; HIC = high-income countries; MIC = middle-income countries.

In bold with + symbol the countries with the highest prevalence of active and sedentary time; in bold with—symbol the countries with the lowest prevalence of active and sedentary time.

**Table 3:** Accelerometer and SIMPAQ measures among socioeconomic development and treatment settings.

	Total	HIC	MIC	F (Main effect*)*	p	Partial Eta Squared *(n*^*2*^*p)*	ES Cohen’s *f*	F Interaction (a, b)	p	Partial Eta Squared *(n*^*2*^*p)*	ES Cohen’s *f*
		Inpatient Outpatient	Inpatient Outpatient								
**Accelerometer MVPA hr/day (SD)**	1.15 (1.08)	1.57 (1.33) 0.81 (0.50)	0.91 (0.96) 0.54 (0.32)	**20.888** ^a^	0.001*	**0.033** ^a^	**0.184**	3.633	0.057	0.006	0.077
				**30.358** ^b^	0.001*	**0.047** ^b^	**0.222**				
**Accelerometer sedentary time hr/day (SD)**	11.36 (2.74)	11.28 (2.75) 10.63 (2.29)	13.62 (2.49) 11.74 (3.02)	**43.004** ^a^	0.001*	**0.066** ^a^	**0.265**	**5.525**	0.019*	**0.009**	**0.095**
				**23.001** ^b^	0.001*	**0.036** ^b^	**0.193**				
				***K***	***p***						
**SIMPAQ MVPA hr/day (SD)**	1.33 (1.26)	1.42 (1.30) 1.29 (1.26)	1.05 (1.22) 1.35 (1.16)	**8.743** ^a^ ^,^ ^b^	0.033*						
**SIMPAQ sedentary time hr/day (SD)**	8.12 (3.31)	7.78 (3.22) 8.31 (3.47)	8.46 (3.28) 8.44 (3.19)	6.587^a^^,^^b^	0.086						

HIC = high-income countries; MIC = middle-income countries; SD = standard deviation

a = socioeconomic development (HIC and MIC)

b = treatment setting (Inpatient and Outpatients). F = ANOVA analysis; ES = Effect size estimated by Cohen *f;* K = Kruskal Wallis tests

* = Statistically significant difference (p ≤ 0.05).

Accelerometry data showed that Brazilian patients exhibited the highest level of SB = 14.24 (SD = 2.4) hours/day, while Italian patients spent the least 8.33 (SD = 1.3) hours/day (Table 2). Portuguese patients reported a longer time of SB in subjective measures 10.00 (SD = 3.2) hours/day, while the least sedentary were Norwegians 6.21 (SD = 2.6) hours/day (Table 2). In the total sample, participants spent 11.36 (SD = 2.74) hours/day on SB according to objective measures (Table 3). However, they self-reported spending 8.12 (SD = 3.31) hours/day on SB (Table 3).

#### Objective and subjective measures among socioeconomic development and treatment setting

*Objective measures (accelerometer)*. ***Physical activity*.** Inpatients from HIC had a mean of 1.57 (SD = 1.33) hours/day of MVPA, while outpatients had a mean of 0.81 (SD = 0.50) hours/day. Inpatients from MIC had a mean of 0.91 (SD = 0.96) hours/day of MVPA, while outpatients had a mean of 0.54 (SD = 0.32) hours/day. In the ANOVA analysis for PA, we found a main effect on socioeconomic development (F = 20.888; p < 0.001; *η*^*2*^*p* = 0.033; small effect size) and on the treatment setting (F = 30.358; p < 0.001; *η*^*2*^*p* = 0.047; small effect size) (Table 3), showing that individuals from HIC performed higher levels of PA than those from MIC, despite the living situation. The magnitude of Cohen’s *f* effect size was also small for socioeconomic development (*f* = 0.184) and the treatment setting (*f* = 0.222) (Cohen, 1988). However, we did not find significant interactions between both variables (F = 3.633; p = 0.057; *η*^*2*^*p* = 0.006). The magnitude of Cohen’s *f* effect size was also small (*f* = 0.077) (Table 3).

#### Sedentary behavior

Inpatients from HIC had a mean of 11.28 (SD = 2.75) hours/day of SB, while outpatients had a mean of 10.63 (SD = 2.29) hours/day. Inpatients from MIC had a mean of 13.62 (SD = 2.49) hours/day of SB, while outpatients had a mean of 11.74 (SD = 3.02) hours/day. The ANOVA analysis showed a main effect on socioeconomic development (F = 43.004; p < 0.001; *η*^*2*^*p* = 0.066; medium effect size) and on treatment setting (F = 23.001; p < 0.001; *η*^*2*^*p* = 0.036; small effect size) (Table 3). The magnitude of Cohen’s *f* effect size was medium for socioeconomic development (*f* = 0.265) and small for treatment settings (*f* = 0.193) (Cohen, 1988). These results showed that individuals from MIC were more sedentary than those from HIC despite the treatment setting, and inpatients were more sedentary than outpatients despite their socioeconomic development. Furthermore, we found a significant interaction between socioeconomic development and treatment setting (F = 5.525; p = 0.019; *η*^*2*^*p* = 0.009; small effect size). Cohen’s *f* effect size was also small (*f* = 0.095) (Cohen, 1988). Furthermore, these results showed that the difference between inpatients and outpatients was greater in MIC, where inpatients were even more sedentary than outpatients compared to HIC.

### Subjective measures (SIMPAQ)

#### Physical activity

Inpatients from HIC self-reported a mean of 1.42 (SD = 1.30) hours/day of active time, while outpatients reported a mean of 1.29 (SD = 1.26) hours/day. Inpatients from MIC had a mean of 1.05 (SD = 1.22) hours/day of active time, while outpatients had a mean of 1.35 (SD = 1.16) hours/day. We found significant differences in favor of patients from HIC (K = 8.743, p = 0.033) (Table 3).

#### Sedentary behavior

Among sedentary time measures, inpatients from HIC had a mean of 7.78 (SD = 3.22) hours/day of SB, while outpatients had a mean of 8.31 (SD = 3.47) hours/day. Meanwhile, inpatients from MIC had a mean of 8.46 (SD = 3.28) hours/day of SB, while outpatients had a mean of 8.44 (SD = 3.19) hours/day. Comparing the HIC, MIC, inpatient, and outpatient groups (K = 6.587; p = 0.086), no significant differences were observed (Table 3).

## Discussion

In a sample with a wide range of diagnoses in 23 countries, our data from objective measures showed that the most physically active patients were from Switzerland, while in subjective measures (SIMPAQ), Japan’s patients were the most physically active in both HICs. In objective measures of sedentary time, most of the sedentary patients were from Brazil, an MIC, while in subjective measures, those from Portugal, an HIC, were the most sedentary. For objective sedentary measures, our main results showed a significant effect on socioeconomic development (showing a medium magnitude of the effect size) and treatment settings (showing a small magnitude of the effect size). Although inpatients were more sedentary than outpatients in both the HIC and MIC, the difference was greater in the MIC, where inpatients were more sedentary. Given the magnitude of the effect sizes found, it seems that socioeconomic development has an even greater influence on the SB levels of these individuals compared to the treatment setting. Furthermore, in subjective sedentary measures, participants from MIC had higher levels of SB, regardless of the treatment setting. However, we did not find significant differences in sedentary time between the socioeconomic development and treatment setting groups. Considering the objective active measures, inpatients were more physically active than the outpatients regardless of socioeconomic development, contrary to our hypothesis. The main effects of socioeconomic development and treatment settings were found; however, the magnitude of the effect size was small for both. In subjective measures, participants from HIC were more physically active in inpatient care, while in those from MIC, lower levels of PA were observed. Moreover, we found significant differences between the socioeconomic development and treatment setting groups in terms of active time.

According to previous evidence, socioeconomic development is inversely associated with SB in participants from HIC, while in those from LMIC, there was a positive association between socioeconomic development and SB [29]. Stubbs and colleagues suggested that among LMIC, sociodemographic factors, such as living in an urban environment, employment status, and male sex, were associated with low levels of PA [30]. Furthermore, they are less likely to meet PA guidelines than individuals from HIC [9]. Despite the difficulties faced by people living in poor countries, stigma and discrimination can also contribute to the treatment gap among individuals with mental health problems [13]. Furthermore, in both participants from HIC and MIC, PA can be incorporated into the daily routine of individuals regardless of diagnosis and can promote psychological well-being across all ages [31].

Considering the aforementioned treatment settings, our results showed that inpatients were more physically active than outpatients in objective measures of both HIC and MIC. Simultaneously, inpatients were more sedentary than outpatients, especially in the MIC, where the difference was greater between the two treatment settings. Similarly to these findings, Vancampfort and colleagues found that inpatients were more physically active than outpatients and those living in the community [9]. Furthermore, it appears that individuals in inpatient care have more access to exercise interventions while at the same time having to stay in bed for longer periods [24]. Outpatients may have a lower chance of participating in exercise interventions, resulting in less moderate to intense activities, but are more likely to commute or spend less time in SB. However, these populations could benefit from exercise programs, as participation in sports results in a decrease in BMI and has been associated with several health benefits [32]. Factors, such as diet, exercise, and lifestyle interventions, can promote important benefits for cardiometabolic outcomes (body weight, blood pressure, and waist circumference) and should be considered [33].

Although there is a growing recognition of the importance of PA in people with mental illness, it is difficult for them to adopt and maintain a physically active lifestyle [34]. These individuals may not have sufficient motivation for PA due to many factors, such as symptomatology, comorbidities, lack of social support, and lack of confidence to engage in and maintain the practice [18, 35]. Despite the recognition of the importance of regular PA for mental and physical health, high rates of physical inactivity worldwide indicate a lack of adequate PA surveillance, policies, and research [36]. Due to geopolitical and socioeconomic factors, health-based models have failed to promote PA in recent decades [37], both in the general population and in populations with mental illnesses. Physical inactivity is a major contributor to the risk of cardiovascular disease [38]. Among individuals with mental illness, the risk of cardiometabolic disease is increased by 1.4–2.0 times compared to individuals without mental illness [31]. Dunstan et al. (2021) highlighted that those who spend long periods sitting in SB and have low levels of PA have a higher risk of all-cause mortality. The authors also showed that an increase in PA levels decreased the risk of all-cause mortality, even in those who spent long periods sitting [38]. In this sense, interventions targeting changes in individual lifestyles through multidisciplinary approaches should be implemented in different treatment settings to mitigate SB and improve active time [31]. Strategies aimed at sitting less and moving more through motivational interviews, along with the implementation of treatment guidelines that contain recommendations for PA, could be an interesting and feasible tool, especially in the context of MIC.

Considering the motivational processes related to the adoption and maintenance of physically active behavior, knowing and understanding autonomous motivation could help increase PA in these populations [39]. In a study involving patients with schizophrenia, major depressive disorder, and bipolar disorder, the authors found a strong association between more autonomous types of motivation and the amount of walking, as well as the MVPA [40]. However, individuals with severe mental illness tend not to meet the PA guidelines for these populations, which recommend 150 min per week of moderate to vigorous intensity [17, 41, 42]. Especially in the MIC, factors such as poor diet, tobacco use, and weak PA national policies are great challenges to maintain a healthy lifestyle [31, 43].

However, this study has some limitations. First, we used a self-reported measure to assess PA among individuals with mental illnesses. Therefore, subjective results may accurately reflect PA and SB patterns and may present some bias. Second, our study included more HICs than MICs, resulting in a large number of participants from HIC. Therefore, the sample size showed great differences in each country due to the particularity of including individuals in different treatment settings and socioeconomic development in the 23 countries involved. Therefore, the sample may not be representative of all countries. Third, for levels of SB, in the ANOVA analysis, the magnitude of effect sizes found that only socioeconomic development showed a medium effect size, while the treatment setting presented a small effect size. Furthermore, for PA levels, the magnitudes of the effect sizes were small in both socioeconomic development and treatment settings. However, the main strength of this study is that we used objective measures to accurately assess PA and SB levels using an accelerometer, presenting concise and accurate data.

Our findings provide results that may be useful to public agencies and policymakers, especially in the context of MICs, as patients living in these countries face more challenges in adopting and maintaining PA practices and reducing sedentary time. Due to the low cost of promotion of PA, this type of intervention could be useful in reducing mental, physical, and social burdens in these countries [15]. Regardless of the socioeconomic development of a country, small changes in the way the patient is approached can contribute to an improvement in an individual’s lifestyle. Non-pharmacological interventions to implement lifestyle approaches may result in a decrease in body weight and may be helpful [44, 45]. In addition, motivational interviews should be conducted to determine individual particularities and needs. As such, the use of a portable activity tracker could be a simple strategy and can promote psychological and physiological benefits by increasing active time [46].

Furthermore, socioeconomic factors may play an important role in the exercise behavior of patients. Without adequate health policies, it is difficult to assess and advise these individuals on how to optimize their PA habits to improve their functional status. In addition, as our main results showed, socioeconomic development also appears to play an important role in the levels of SB of individuals with mental illness, warning of the need to develop new strategies to reduce these levels in this population. However, the multifactorial etiology of mental illness must be considered, and the need for multidisciplinary physical health personnel could be a useful tool to motivate behavioral changes in these populations [47]. Furthermore, adequate training of physical and mental health staff is needed to address behavioral change techniques, improve lifestyle interventions, and mitigate healthy and physically active behaviors. Despite the lack of primary healthcare research in the MIC and the lack of evidence, our study is the first to reveal discrepancies between countries and settings for those with mental illness.

## Conclusion

In conclusion, our findings could support novel strategies to reduce SB and support a physically active and healthy lifestyle in individuals with mental illness in these countries, thus filling this gap. In this sense, knowing the socioeconomic barriers that could affect PA and SB can be an important tool to increase overall PA and address the mental health needs of each country in a way that prioritizes the transformation of health systems.

## Supporting information

S1 Checklist STROBE statement—Checklist of items that should be included in reports of cross-sectional studies. (DOC)
