# Correction: Physical activity and sedentary behavior levels among individuals with mental illness: A cross-sectional study from 23 countries

**DOI:** 10.1371/journal.pone.0316870

**Published:** 2024-12-30

**Authors:** Fernanda Castro Monteiro, Felipe de Oliveira Silva, Aline Josiane Waclawovsky, José Vinícius Alves Ferreira, Fabianna Resende de Jesus-Moraleida, Felipe Barreto Schuch, Philip B. Ward, Simon Rosenbaum, Rachel Morell, Lara Carneiro, Andrea Camaz Deslandes

The affiliation for the sixth author is not indicated. Felipe Barreto Schuch is also affiliated with: Faculty of Health Sciences, Universidad Autónoma de Chile, Providencia, Chile.
